# Puerperal Toxemia

**Published:** 1907-10

**Authors:** 


### PUERPERAL TOXEMIA.

William H. Wells believes that the exact causes of toxemia in pregnancy are yet obscure. Its treatment is based upon efforts to increase elimination, first of all by the bowels. Purgation should be started and continued until the bowels are absolutely clean, and a good flow of that natural intestinal antiseptic, the bile, is started. For this purpose, calomel given in doses of one-fourth grain every hour until 10 doses are taken, and often combined with one-fourth to one-half drop of croton oil, and followed by the free use of salines, gives in his experience the best results. If there is vomiting, lavage of the stomach may be practiced with good effect, the stomach being washed out with sterile water with bicarbonate of soda, one dram to the quart. Unload the lower bowel by washing out the colon, first by the usual "triplex enema," then by lavage of normal salt solution. The lavage should be repeated three or four times a day until the solution comes out clear. Following the enteroclysis, intestinal peristalis should be stimulated by such drugs as nux vomica, cascara, and possibly aloes, while a mild impression of calomel may be kept up for some time after the attack is over. Strict attention should be given to the bowels until the end of pregnancy. Excretion through the skin should be stimulated in mild cases of toxemia by simple warm baths, proper clothing and exercise. In more severe cases, the hot pack will be necessary. To quiet the high tensioned circulation, bromide or chloral in suitable doses may be used, or the tincture of veratrum viride in doses of 15 or 20 minims is better. When the nervous tension is high a hypodermic injection of one-quarter grain of morphin does good and may be repeated once. It quiets the restlessness until the eliminatives have time to work. To increase the flow of urine and favor excretion, about two quarts of water should be taken in the 24 hours, sweet spirits of niter, citrate of sodium or lithium, acetate of potassium and salicylate of sodium are all of use. In many cases the toxemia will respond to treatment, and the interruption of the pregnancy will be unnecessary. The earlier treatment is started, the better the prognosis. In the same Journal, John Cooke Hirst states, concerning puerpal eclampsia, that the place for an ecclamptic is in a hospital ,treatment in a private house adding considerably to the danger. He gives as a routine method of treatment the following: first avert the convulsion if possible with chloroform, and prevent injury to the tongue during convulsions by a mouth gag of some kind; second, give 15 minims of tincture veratrum viride hypodermically; 3rd, wash out the stomach, and through the tube pour in two ounces of castor oil, or place on back of tongue four drops of croton oil and ten drops sweet oil; fourth, hot vapor bath or hot pack for thirty minutes every four hours; fifth, hypodermicilysis of one pint of salt solution under breast every eight hours: sixth, if convulsions recur repeat veratrum viride in five minim doses every hour and if after three hours pulse is still bounding and patient cyanosed, perform venesection; seventh, under ordinary circumstances let labor alone; should a patient recover from eclampsia during the middle trimester or later, labor should be induced in the eight month of pregnancy.—Therapeutic Gazette, April, 1907.—Cleveland Med. Journal.

